# Motion artifact removal from photoplethysmographic signals by combining temporally constrained independent component analysis and adaptive filter

**DOI:** 10.1186/1475-925X-13-50

**Published:** 2014-04-24

**Authors:** Fulai Peng, Zhengbo Zhang, Xiaoming Gou, Hongyun Liu, Weidong Wang

**Affiliations:** 1 School of Information and Electronics, Beijing Institute of Technology, Beijing, China; 2 Department of Biomedical Engineering, Chinese PLA General Hospital, Beijing, China

**Keywords:** Photoplethysmographic signal, Motion artifact, Independent component analysis, Adaptive filter

## Abstract

**Background:**

The calculation of arterial oxygen saturation (SpO_2_) relies heavily on the amplitude information of the high-quality photoplethysmographic (PPG) signals, which could be contaminated by motion artifacts (MA) during monitoring.

**Methods:**

A new method combining temporally constrained independent component analysis (cICA) and adaptive filters is presented here to extract the clean PPG signals from the MA corrupted PPG signals with the amplitude information reserved. The underlying PPG signal could be extracted from the MA contaminated PPG signals automatically by using cICA algorithm. Then the amplitude information of the PPG signals could be recovered by using adaptive filters.

**Results:**

Compared with conventional ICA algorithms, the proposed approach is permutation and scale ambiguity-free. Numerical examples with both synthetic datasets and real-world MA corrupted PPG signals demonstrate that the proposed method could remove the MA from MA contaminated PPG signals more effectively than the two existing FFT-LMS and moving average filter (MAF) methods.

**Conclusions:**

This paper presents a new method which combines the cICA algorithm and adaptive filter to extract the underlying PPG signals from the MA contaminated PPG signals with the amplitude information reserved. The new method could be used in the situations where one wants to extract the interested source automatically from the mixed observed signals with the amplitude information reserved. The results of study demonstrated the efficacy of this proposed method.

## Background

Pulse oximeter has been widely utilized to measure the level of arterial oxygen saturation (SpO_2_) and pulse rate (PR) of humans noninvasively. It is based on the principles: 1) the different light absorption properties between oxyhemoglobin (HbO_2_) and deoxyhemoglobin (Hb); 2) only the arterial blood (provided that the mildly pulsatile venous blood can be neglected) pulsate in the tissue contributing to the pulsation of emergent light intensity (termed *AC part*), while others correspond to the emergent light intensity baseline (termed *DC part*). Generally, a pulse oximeter employs double wavelengths of light (red and infrared (IR)) for the emission sources and a photodiode as detector to receive the information-bearing light from the same or the opposite side with respect to the emitter. The measurement positions of pulse oximeter are usually fingertips, earlobes, toes, foreheads, etc., since the capillary network of these parts are abundant. A pulse oximeter is precise provided with clean PPG signals, which are related to the blood volume changes in the microvascular bed of tissue [1]. However, it is not a trivial task to acquire interference-free clean PPG signals in real-world applications. Numerous factors, such as MA, ambient lights, low perfusion and temperature variations could lead to pulse oximeters’ performance degradation. In particular, the removal of MA, which is caused by voluntary or involuntary movements of the individual during the measurement, is always challenging ever since the appearance of pulse oximeters. Conventional filters are incapable to get rid of MA effectively due to the frequency overlaps between the MA and clean PPG signal [2]. Researchers have developed numerous approaches to tackle this issue. The MAF method is good at suppressing the sporadically occurring noise in the corrupted PPG signals, while it is at its wit’s end before strong or sudden occurring artifacts [3]. Adaptive filters, which could adjust their weight vector based on adaptive algorithms, are powerful tools to deal with the in-band noise, provided that the reference signal (which is either correlated with the MA part but uncorrelated with PPG signal or correlated with the clean PPG signal but uncorrelated with the MA) is available. One way to obtain the reference signal is with the help of extra hardware such as accelerometers [4-7] or photoelectric devices [8]. Another way is to synthesize the reference signal from the two channel contaminated PPG signals [2,9-11]. In consideration of the nonstationarity of PPG signal, wavelet transform is performed to remove MA [12-14]. The empirical mode decomposition (EMD), which is another powerful decomposition to handle non-stationary signal, has been studied in [15,16]. Although these two methods could reduce the MA to some extent, both of them are troubled with the problem: how to select an appropriate threshold to decide which components should be removed. High order statistics are used in [17] to extract clean artifact-free PPG signals preserving all the essential morphological features required. Applying cycle-by-cycle Fourier series analysis (CFSA) to deal with MA also demonstrates a satisfying performance [18]. However, the period of every PPG signal cycle must be acquired precisely when applying CFSA method. Based on the independence between the PPG signal and the MA, ICA combining a signal enhancement preprocessor is used to separate the PPG signal from the contaminated original PPG signal [19], from which the efficacy of the ICA algorithm in dealing with the MA corrupted PPG signals could be confirmed. Despite the excellent performance of the ICA method, one must keep in mind that the ICA has permutation and scale ambiguities [20]. Meanwhile, the SpO_2_ computation needs the accurate amplitude information of both the red and IR light channel PPG signals, the ICA output cannot be used to calculate the SpO_2_ value directly.

In this paper, we introduce a new method combining cICA [21] and adaptive filters to deal with the aforementioned problems related to ICA. By using cICA, we could obtain the interested component automatically. By using the adaptive filter, we could effectively remove the MA with the PPG signal amplitude information recovered. In this paper’s method, we firstly extract the artifact-free PPG-correlated component from the contaminated measured PPG signal by cICA, then pass the output of the cICA through the adaptive filters to obtain the two channel artifact-free PPG signals with the amplitude information reserved. In order to evaluate the efficiency of our method, FFT-based MA removal algorithm proposed in [2] and MAF method were used as comparisons. Experiments with synthetic and real-world data were performed to demonstrate the efficacy of the proposed method.

## Methods

### Mathematical preliminaries

#### Constrained independent component analysis

ICA can be used to separate the observed mixed signals (**X**) into several independent sources (**S**) based on certain criteria, such as maximization of non-Gaussianity, minimization of mutual information and maximum likelihood estimation. The relationship between the observed signals (**X**) and independent sources (**S**) can be expressed as a linear mixture:

(1) X=AS

where **A** is the unknown mixing matrix. The independent sources **S** can be obtained when finding an unmixing matrix **W** (=**A**^− 1^), as:

(2) S=WX

Several different implementations of ICA can be found in [22-25].

Because both **A** and **S** in equation (1) are unknown, ICA has the permutation ambiguity that has been mentioned in the introduction section. Where one desires a specific IC, the cICA presented in [21] can effectively extract the desired component incorporating with a reference signal. The cICA algorithm can be modeled as:

(3) $$\begin{array}{l} \mathrm{Maximize}:J\left(y\right)\approx \rho {\left[E\left\{G\left(w^{T}x\right)\right\}\;-\;E\left\{G\left(v\right)\right\}\right]}^{2}\\ \mathrm{Subject}\;\mathrm{to}:g\left(w\right)=\epsilon \left(y,r\right)\;-\;\xi \leq 0,\;h\left(w\right)=E\left\{y^{2}\right\}\;-\;1=0 \end{array}$$

where J(y) is the approximate negentropy, *ρ* is a positive constant, G(⋅) can be any non-quadratic function, v is a zero mean, unit variance Gaussian variable, g(**w**) is the closeness constraint, *ϵ*(y, r) is the closeness measure, *ξ* is the closeness threshold and the equality *h*(**w**) is to ensure that the contrast function J(y) and the weight vector **w** are bounded. Reference [21] presents a solution for the problem of (3), where they considered it as a constraint optimization problem. By using the Newton-like learning method the optimum weight vector **w** for the desired signal could be found.

#### Adaptive filter algorithm

For simplicity, the adaptive algorithm we used in this paper is the Least Mean Square (LMS) algorithm:

(4) $$\begin{array}{l} \;y\left(n\right)=w^{T}\left(n\right)u\left(n\right)\\ e\left(n\right)=d\left(n\right)\;-\;y\left(n\right)\\ w\left(n+1\right)=w\left(n\right)+\mu e\left(n\right)u\left(n\right) \end{array}$$

where **u**(n) is the filter input, which could be either the MA part or the PPG signal part, d(n) is the desired signal, which is the weighted summation of the MA part and PPG signal, **w**(n) is the weight of the filter, e(n) is the error induced by the adaptive filter and *μ* is the step size used in weight vector update.

#### Fast Fourier transform (FFT) combining LMS (FFT-LMS) method

In this section we briefly introduce the FFT-LMS method described in [2] as a comparison to our method. The steps of the FFT-LMS method are as follows:

a) After applying FFT on the MA contaminated PPG signal, the frequency spectra of three different parts in the corrupted PPG signal are obtained: pulsatile PPG portion (0.5-4 Hz), respiratory activity (0.2-0.35 Hz) and MA component (0.1 Hz or more).

b) The coefficients of the frequency component corresponding to the pulsatile PPG portion and respiration component are set to zero to generate the MA reference. Thus, a modified frequency spectrum corresponding to MA noise is obtained.

c) By applying the inverse FFT on the modified spectrum, a synthetic noise reference signal in time-domain is generated.

d) The synthetic MA noise is then fed into the LMS adaptive filter as the reference signal, with the MA corrupted PPG signal acting as the desired signal.

### Motion artifact removal by combining cICA and adaptive filter

The red and IR channel signals (**X**) can be modeled as the linear mixture of MA and PPG signal sources (**S**). The MA signal is postulated as the complex combination of multiple sources, which means that the measured signals may contain more than two independent sources [19]. Unlike the conventional ICA algorithm, the cICA algorithm, which needs no assumption regarding the number of actual underlying sources could automatically extract a specific source. Due to the fact that the PPG signal possesses periodic behaviour, the PPG-correlated component could be extracted by using the cICA algorithm, with the help of the periodic information of PPG signal. However, this obtained PPG-correlated component misses the amplitude information.

The adaptive filter can remove the in-band MA noise effectively provided that the reference input which should be correlated with either MA component or PPG component has been obtained. In our study, we combine the cICA algorithm and adaptive filter to remove the MA from PPG signals. On one hand, the adaptive filter can recover the amplitude information of the PPG-correlated component obtained by the cICA algorithm. On the other hand, the PPG-correlated component can serve as the reference input for the adaptive filter.

**Figure 1:**
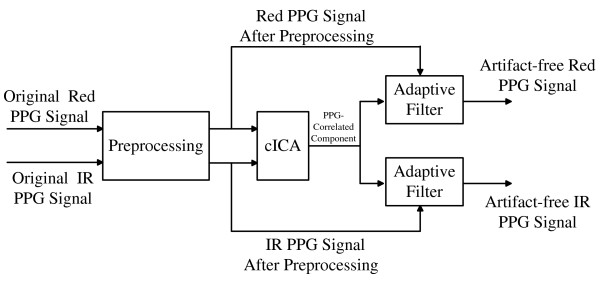
The primary flow chart of the proposed method.

The main idea of the method is described briefly in Figure 1. To test the effectiveness of our approach in extracting the underlying PPG component from MA corrupted PPG signals, we validate our approach in the following sections.

#### Preparation for the proposed method

##### Low-pass filtering and DC removal

The signals captured by the signal-acquisition instrument inevitably contain plenty high frequency noise which is the mixture of the ambient light induced noise, thermal noise, electromagnetic noise especially the power frequency interference (50/60 Hz) and other unclassified noise. Fortunately, these noise usually possess the characteristic of either wide band frequency spectra or higher frequency contrasted to the PPG signal, such that a conventional low-pass filter could be utilized to remove the bulk of noise. Based on the fact that PPG signal frequency distributes within the range 0.5-4 Hz [2], we use a FIR hamming window low-pass filter with 20 dB attenuation at 8 Hz to wipe out most of the high frequency noise.

To separate the DC part, a first-order IIR filter is used, the transfer function is:

(5) $$\;H\left(z\right)=\frac{Y\left(z\right)}{X\left(z\right)}=\frac{1\;-\;z^{-1}}{1\;-\;0.992z^{-1}}$$

which could provide an attenuation about 20 dB for DC component and have negligible effect on the AC part.

##### Generating the reference signal for cICA

To generate the reference signal for cICA, we must obtain some prior knowledge about the desired signal. Generally, the period of the desired signal is the straightforward information to generate the reference signal. Since that PPG signal exhibits periodic behaviour and MA component is mainly caused by voluntary or involuntary movements which result in irregular waveform, we can use the periodic information of the PPG signal to generate the reference signal. Autocorrelation is implemented on the MA corrupted PPG signal to obtain the period of the PPG signal after the low-pass filtering and DC removal process. A reference signal with periodic rectangular pulse waves is generated based on the periodic information obtained from the original PPG signals.

#### Detailed implementation of the new method

**Figure 2:**
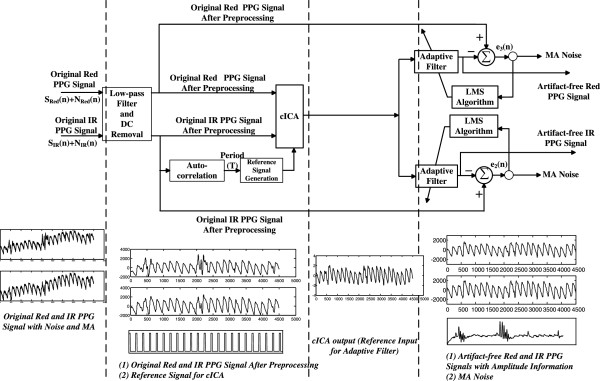
Detailed block diagram of the proposed MA removal scheme.

The whole detailed block diagram of our approach is shown in Figure 2, which is the refinement of Figure 1.

The detailed steps of the proposed method are described as follows:

a) The two channel original PPG signals are firstly processed by the filters described above to remove the high frequency noise and the DC component.

b) Autocorrelation is implemented on the IR channel signal to get the period of the PPG signal, and the reference signal for cICA is generated based on the period obtained.

c) The two channel preprocessed PPG signals and the reference signal generated in step b are all fed into the cICA algorithm. Then the artifact-free PPG-correlated component is generated.

d) The artifact-free PPG-correlated component is fed into the adaptive filter as the reference input to recover the amplitude information of the two channel PPG signals. The two channel corrupted PPG signals act as the corresponding desired signals respectively. Two channel MA-reduced PPG signals are obtained.

For simplicity, the LMS algorithm is used here. In the next context, we name our method as cICA-LMS.

### Experiments setup

#### Synthetic dataset simulation

In this section, a simulation experiment was performed on the synthetic dataset by using the cICA-LMS, FFT-LMS and MAF methods. In the simulation, we mixed two signals: the target PPG signal (s(t)), which was captured from the stationary finger, and another, unwanted signal (MA(t)), which was *randomly generated* from the MA corrupted PPG signals. The two signals were synthesized in the following way:

(6) $$x\left(t\right)=s\left(t\right)+\lambda \mathrm{MA}\left(t\right)$$

where x(t) is the mixed signal, and parameter *λ* defines the proportion of MA(t) in x(t). Changing parameter *λ* alters the ratio of MA noise part to PPG signal in the mixed signal. In order to investigate the efficiency of our method with respect to different MA noise proportions occupied in the mixed signal, we used parameter SNR defined as:

(7) $$\mathrm{SNR}=20\log\frac{\mathrm{RMS}\left(s\left(t\right)\right)}{\mathrm{RMS}\left(\lambda \mathrm{MA}\left(t\right)\right)}\;\mathrm{dB}$$

The simulation performance is expressed in terms of relative root mean squared error (RRMSE), which is defined as:

(8) $$\mathrm{RRMSE}=\frac{\mathrm{RMS}\left(s\left(t\right)-\hat{s}\left(t\right)\right)}{\mathrm{RMS}\left(s\left(t\right)\right)}100\left[\%\right]$$

where $\hat{s}\left(t\right)$ is the estimation of the interested signal s(t).

#### Real-world MA corrupted PPG signals

To validate cICA-LMS method in removing MA from corrupted PPG signals, we considered five different motion situations. Seven healthy volunteers (four males with average age (29 ± 6) and three females with average age (25 ± 2)) were recruited to perform the experiments with the informed consent obtained. The procedure was also approved by the Ethics Committee of Chinese PLA General Hospital. Before the experiment, all the participants were required to sit still within doors (18 Celsius degree) for five minutes. The PPG signals were captured from the index finger using AFE4400SPO2EVM (from Texas Instruments Corporation) with 200 Hz sample frequency. Five different motions (vertical movement of finger, horizontal movement of finger, bending finger, pressurizing probe clip and waving hand) were performed during data acquisition. Each recording consists of six different sections: 1-min motionless period, 1-min vertical movement period, 1-min horizontal movement period, 1-min bending finger period, 1-min pressurizing probe clip period and 1-min waving hand period.

## Results

### Synthetic dataset

**Figure 3:**
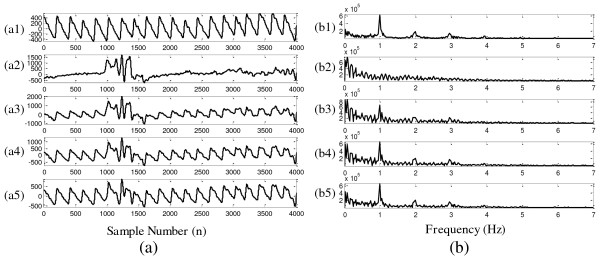
**Synthetic signals with different SNRs. (a1)**: Clean PPG signal- s(t); **(a2)**: MA noise- MA(t); **(a3)-(a5)**: Mixed signals- x(t) with SNR equaled to −5 dB, 0 dB, 5 dB respectively; **(b1)-(b5)**: Frequency spectrum corresponding to **(a1)-(a5)**.

Figure 3 shows the waveforms of the clean PPG signal, the real MA noise part, and three mixed signals with SNRs of −5 dB, 0 dB and 5 dB. The corresponding frequency spectra of different signals are also provided in the right sub-figure. It is very clear that the frequency distribution of the MA and PPG signal are overlapped. Therefore, the conventional filter with constant cut-off frequency cannot reduce the MA effectively.

**Figure 4:**
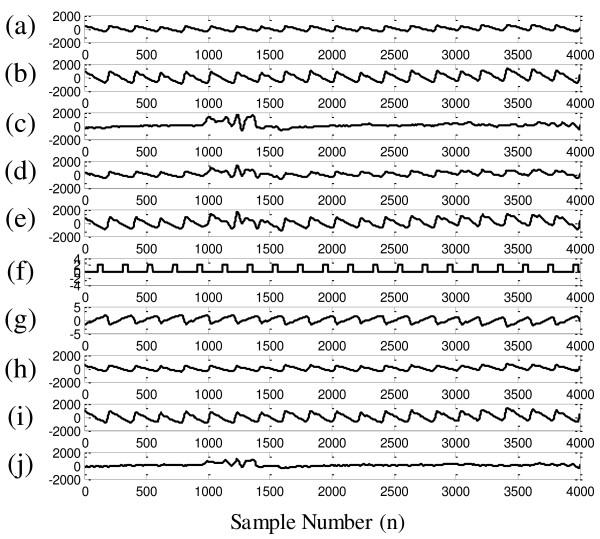
**Visual performance of the proposed method in extracting the interested signal from synthetic signals. (a)**: Clean red PPG signal; **(b)**: Clean IR PPG signal; **(c)**: MA noise; **(d)**: Mixed signal of the red PPG signal and MA noise with SNR = 0 dB; **(e)**: Mixed signal of the IR PPG signal and MA noise with SNR = 0 dB; **(f)**: Reference signal for the cICA; **(g)**: cICA output; **(h)**: Recovered red PPG signal; **(i)**: Recovered IR PPG signal; **(j)**: Recovered MA noise by cICA-LMS algorithm.

Figure 4 presents the waveforms of the cICA-LMS method applying on the synthetic signal with SNR equaled to 0 dB. Figure 4a-b are 20-s epoch of red channel and IR channel PPG signals captured from the stationary fingertip. Figure 4c is the MA noise component extracted from the MA corrupted PPG signal by the cICA-LMS method. Figure 4d-e are the linear mixed signals of the two channel PPG signals and the MA with SNR equaled to 0 dB. Figure 4f is the reference signal for the cICA algorithm generated from the mixed IR PPG signal. It can be seen that the reference signal have the same period with the PPG signal. Figure 4g is the output of the cICA, i.e. artifact-free PPG-correlated component, which loses the energy information of the original PPG signal. Figure 4h-j are the artifact-free red and IR PPG signals and the MA noise extracted from the corrupted PPG signals, respectively. Both the red and IR PPG signals are effectively separated from the MA with the amplitude information reserved.

**Figure 5:**
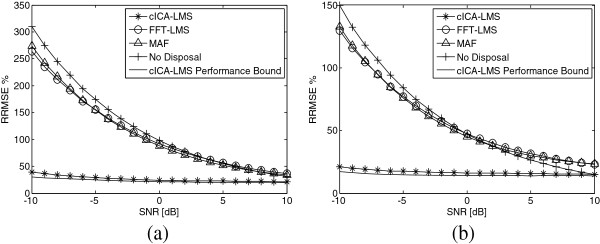
**Statistic performance of cICA-LMS method in comparison with FFT-LMS and MAF methods.** It shows the RRMSE (%) changing with SNR (dB) of three different methods. The performance bound of cICA-LMS is also shown using the solid line. **(a)**: Red channel PPG; **(b)**: IR channel PPG.

Figure 5 presents the mean result of 100 times Monte Carlo simulations on the synthetic signals by using the cICA-LMS method. The results of the FFT-LMS and MAF methods are also provided for comparison. In each of the sub-figure, the x-coordinate is the SNR changing from −10 dB to 10 dB with a step size of 1 dB, and the y-coordinate is the RRMSE. In addition, we roughly took the best performance among 1000 times Monte Carlo simulations as the performance bound for the proposed method, which is also shown in the figure.

### MA removal from the real-world corrupted PPG signals

**Figure 6:**
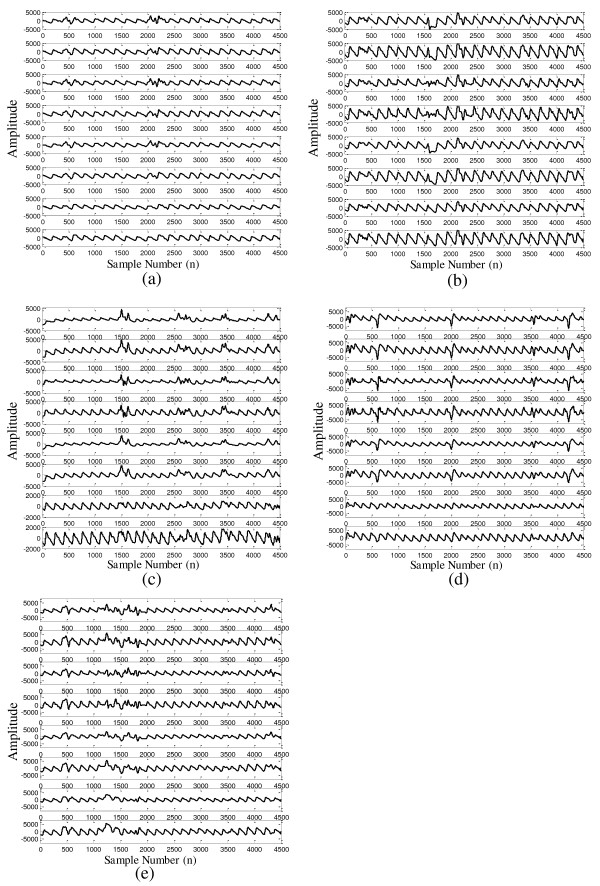
**Performance of the cICA-LMS in reducing the MA under five different motion situations. (a)**: Vertical movement of finger; **(b)**: Horizontal movement of finger **(c)**: Bending finger; **(d)**: Pressurizing probe clip; **(e)**: Waving hand. In each of the sub-figure, the upper two subplots are MA corrupted red and IR channel PPG signals, the next two subplots are recovered PPG signals using FFT-LMS method, then the next two subplots are recovered PPG signals using MAF method, and the last two subplots are recovered PPG signals by the cICA-LMS method.

Figure 6 shows the results of the cICA-LMS method in dealing with MA under five different motion situations: vertical movement of the finger (Figure 6a), horizontal movement of the finger (Figure 6b), bending the finger (Figure 6c), pressurizing probe clip (Figure 6d) and waving hand (Figure 6e). The performance of FFT-LMS and MAF methods are also shown in the figure for comparison. In each of the sub-figure, the upper two subplots are the red and IR PPG signals corrupted by MA, the next two subplots are MA reduced signals by the FFT-LMS method, then the next two subplots are MA reduced signals by MAF method, and the last two subplots are MA reduced signals by the proposed cICA-LMS method.

As the calculation of SpO_2_ depends on the peak-to-peak values of PPG signals, it is important that the MA reduction method proposed here preserves this characteristic in the recovered PPG. We used the peak-to-peak values of PPG signals to evaluate the efficacy of our method [18]. Table 1 shows the results of the peak-to-peak values (in terms of mean ± standard deviation) of corresponding MA reduced red channel PPG signal by the three methods. The PPG signal without MA and the PPG signal corrupted by MA are also showed for contrast. Similarly, Table 2 shows the results of corresponding peak-to-peak values of IR channel PPG signal. The values in the tables are dimensionless, this does not influence the calculation of SpO_2_.

**Table 1:** Effectiveness of the proposed method in restoring the peak-to-peak values of red channel PPG

	**Vertical movement**	**Horizontal movement**	**Bending finger**	**Pressurizing probe clip**	**Waving hand**
**PPG without MA**	7009.0 ± 408.3	4377.5 ± 435.8	792.7 ± 215.8	2843.4 ± 411.1	2708.0 ± 428.1
**PPG with MA**	6247.8 ± 840.8	4501.6 ± 886.0	1805.4 ± 1076.2	3890.1 ± 2020.7	3278.4 ± 821.1
**Recovered PPG Using FFT-LMS**	6412.6 ± 1058.3	4159.3 ± 1018.9	1836.7 ± 1029.0	3587.8 ± 1494.2	3224.3 ± 894.5
**Recovered PPG Using MAF**	5431.1 ± 939.5	4098.5 ± 856.4	1549.4 ± 846.2	3216.7 ± 1440.6	2855.4 ± 642.8
**Recovered PPG Using cICA-LMS**	6284.6 ± 606.3	4196.2 ± 688.9	971.2 ± 338.1	3181.4 ± 473.4	2735.3 ± 589.5

Data are expressed as mean ± SD. The values in the tables are dimensionless, this does not influence the calculation of SpO_2_.

**Table 2:** Effectiveness of the proposed method in restoring the peak-to-peak values of IR channel PPG

	**Vertical movement**	**Horizontal movement**	**Bending finger**	**Pressurizing probe clip**	**Waving hand**
**PPG without MA**	8294.3 ± 534.8	6502.2 ± 703.0	1754.1 ± 367.4	4218.9 ± 642.5	3886.2 ± 602.0
**PPG with MA**	8204.5 ± 931.4	6728.7 ± 1072.8	2879.1 ± 1028.0	5293.5 ± 1888.5	4549.3 ± 829.2
**Recovered PPG Using FFT-LMS**	8316.7 ± 1232.4	6235.9 ± 1376.3	2674.8 ± 721.2	4941.6 ± 1325.9	4464.9 ± 1057.8
**Recovered PPG Using MAF**	7238.4 ± 1057.3	5824.3 ± 1167.4	2485.8 ± 817.2	4556.4 ± 1353.5	3970.6 ± 816.2
**Recovered PPG Using cICA-LMS**	8091.4 ± 780.5	6299.5 ± 835.9	2129.2 ± 258.0	4806.2 ± 942.1	4125.6 ± 888.9

Data are expressed as mean ± SD. The values in the tables are dimensionless, this does not influence the calculation of SpO_2_.

## Discussion

The results of both the synthetic datasets and real-world experiments demonstrated that the proposed cICA-LMS algorithm could remove MA component from PPG signals effectively. The results also indicate that the cICA-LMS method outperforms the FFT-LMS and MAF methods. Unlike the FFT-LMS method, the cICA algorithm could effectively produce the reference signal for adaptive filter without any assumption of the frequency distribution. Our method could deal with the in-band MA noise effectively when MA noise and PPG signal are independent. In the simulation section, the cICA-LMS algorithm performed very well to extract the sources from the mixed signals. This excellent performance is due to fact that the synthetic mixed signals are from a linear summation of two completely independent components. In the situations where the cICA-LMS is applied on the real-world MA corrupted PPG signals, the cICA-LMS algorithm may not perform as well as that in the simulation dataset. This might be because that the MA component is produced by a quite complex mechanism, and is not completely independent from the PPG signals. Even though, the cICA-LMS algorithm still presents much better performance than the FFT-LMS and MAF methods. The compromised performance of the FFT-LMS and MAF methods may be caused by the frequency overlap between MA component and PPG signal.

Although the use of ICA to remove MA from the corrupted PPG signals has exhibited good result [19], the extracted components by conventional ICA are actually not ordered. Hence, in our study, the cICA algorithm is proposed to deal with this problem. For signal recordings which have large numbers of channels, the cICA algorithm could avoid the subsequent laborious and highly subjective analysis on the large number of resulting extracted sources. To the best of our knowledge, the cICA has not been used to extract the underlying PPG signal from the MA corrupted PPG signals previously. Another inherent disadvantage of conventional ICA is that the extracted components do not include amplitude information of the original sources. Because the ultimate goal of our work is to extract clean PPG signals for SpO_2_ calculation, amplitude information of the extracted PPG signal is indispensable. In order to recover the amplitude information of the extracted PPG signal, an adaptive filter is used in our method with the output of the cICA as reference signal. The results demonstrated that the algorithm combing cICA and adaptive filter performed very well both in the synthetic dataset and real-world PPG signals experiments.

The excellent performance of our method in extracting the underlying PPG signal from the MA corrupted signals lies in the assumption that the MA and PPG signals are independent. In the situations where the MA and PPG signals are not independent, the performance of our algorithm might be compromised. In addition, generating the correct reference signal for cICA plays an important role in extracting the source of interest. In situations where the MA possesses the same period with the PPG signal, the cICA algorithm may fail to produce correct output. However, this does not happen frequently in real life.

The adaptive algorithm used in this study is the LMS algorithm. For LMS algorithm, the step size is very critical in controlling the stability and convergence speed of the algorithm. Improper step size may degrade the performance of our cICA-LMS algorithm. In order to improve the characteristics of the LMS-based adaptive filter, some other algorithms could be tried, such as RLS, NLMS and any other suitable algorithms.

## Conclusions

This paper presents a new method which combines the cICA algorithm and adaptive filter to remove MA component from the MA contaminated PPG signals with the amplitude information reserved. The contribution of this paper lies in the fact that the new algorithm has solved permutation and scale ambiguity problems of conventional ICA. Thus, this algorithm could be used in the situations where one wants to extract the interested source automatically from the mixed observed signals with the amplitude information reserved. The results of this study demonstrated the effectiveness of this proposed method.

## Abbreviations

SpO2: Arterial oxygen saturation; PPG: Photoplethysmographic; MA: Motion artifact; MAF: Moving average filter; ICA: Independent component analysis; cICA: Constrained independent component analysis; FFT: Fast Fourier transform; IR: Infrared; SNR: Signal noise ratio; RRMSE: Relative root mean squared error; LMS: Least mean square; NLMS: Normalized least mean square; RLS: Recursive least square.

## Competing interests

The authors declare that they have no competing financial interests.

## Authors’ contributions

FP: proposed the new method, conducted the simulation and clinical experiments and drafted the manuscript; ZZ: gave a careful proofread to correct those grammar and usage errors; XG: revised the framework and gave a careful proofread to correct those grammar and usage errors; HL: carried on the simulation and clinical experiments and acquired experimental data; WW: have been involved in revising the manuscript critically for important intellectual content and have given final approval of the version to be published. All authors read and approved the final manuscript.
